# Effects of restrictions on maternal feed intake on the immune indexes of umbilical cord blood and liver Toll-like receptor signaling pathways in fetal goats during pregnancy

**DOI:** 10.1186/s40104-019-0336-7

**Published:** 2019-04-16

**Authors:** Wenxun Chen, Qiongxian Yan, Hong Yang, Xiaoling Zhou, Zhiliang Tan

**Affiliations:** 1 CAS Key Laboratory for Agro-Ecological Processes in Subtropical Region, National Engineering Laboratory for Pollution Control and Waste Utilization in Livestock and Poultry Production, Hunan Provincial Engineering Research Center for Healthy Livestock and Poultry Production, South-Central Experimental Station of Animal Nutrition and Feed Science in Ministry of Agriculture, Institute of Subtropical Agriculture, Chinese Academy of Sciences, Changsha, Hunan 410125 People’s Republic of China; 2 University of Chinese Academy of Science, Beijing, 100049 People’s Republic of China; 3 College of Animal Science, Tarim University, Alaer, 843300 People’s Republic of China; 4 Hunan Co-Innovation Center of Animal Production Safety, CICAPS, Changsha, Hunan 410128 People’s Republic of China; 5 Institute of Subtropical Agriculture, Chinese Academy of Sciences, Changsha, Hunan People’s Republic of China

**Keywords:** Feed intake restriction, Fetal goats, Immune cell, Liver, TLRs signaling pathway

## Abstract

**Background:**

Liver has important immune function during fetal development and after birth. However, the effect of maternal malnutrition on immune function of the fetal liver is rarely reported. In this study, twelve pregnant goats (Xiangdong black goat, at d 45 of gestation) were assigned to the control group (fed 100% of nutritional requirements) and the restriction group (fed 60% of the intake of the control group) during gestation from d 55 to 100. Fetal goats were harvested at d 100 of gestation and immune indexes and amino acid profiles of the umbilical cord blood and liver Toll-like receptors (TLRs) signaling pathways were measured.

**Results:**

Maternal body weight in the restriction group was lower than the control group (*P* < 0.05). Maternal feed intake restriction decreased (*P <* 0.05) heart weight, heart index, alkaline phosphatase and serum amyloid protein A in the umbilical cord blood (UCB). Moreover, only histidine was decreased in the restricted group (*P* = 0.084), and there were no differences in other amino acids contents in the UCB between the two groups (*P* > 0.05). The *TLR2* and *TLR4* mRNA expression in the fetal liver in the restriction group was greater (*P <* 0.05) than that in the control group. Furthermore, the mRNA expression levels of myeloid differentiation primary response 88 (*MyD88*), TNF receptor associated factor 6, nuclear factor kappa B subunit 1, NFKB inhibitor alpha, *IFN-β*, *TGF-β*, *TNF-α* and *IL-1β* in the restricted group were upregulated (*P <* 0.05), and the expression of *TLR3* (*P =* 0.099) tended to be higher in the restricted group. However, protein levels of TLR2, TLR4, IκBα, phosphorylated IκBα, phosphorylated IκBα/total IκBα, TRIF and MyD88 were not affected (*P >* 0.05) by maternal intake restriction.

**Conclusions:**

These results revealed that the restriction of maternal feed intake influenced the development of heart and hepatic protein synthesis at the acute phase of fetal goats and upregulated the mRNA expression of genes involved in MyD88-dependent signaling pathways and of target cytokines.

## Background

Maternal malnutrition during pregnancy often leads to intrauterine growth restriction (IUGR) where some visceral organs of IUGR fetuses experience structural changes and impaired function. Epidemiological studies have shown that adult diseases, such as abdominal adiposity, hypertension, dyslipidemia, glucose intolerance, type 2 diabetes and cardiovascular disease are associated with low-birth weight [3, 4]. Fetal malnutrition usually induces permanent anatomical and functional changes in various tissues and organs [18, 20]. These early insults at critical stages of development often lead to permanent changes in tissue structure and function for mammals; this process is known as intrauterine programming [14]. Meanwhile, it is also confirmed that organisms have the ability to change structure and function in response to environmental signals; this process is called developmental plasticity [16].

The liver has multiple important physiological functions during embryonic development and after birth including hematopoiesis, metabolism and immunity. The liver has a unique vascular system that receives blood from the digestive tract through the portal vein so that the liver is continuously exposed to the environment of pathogenic microorganisms [28]. Continuously stimulated by these gastrointestinal antigens, the liver forms a unique immune response through series of pattern recognition receptors, primarily Toll-like receptors (TLRs). Many kinds of cells in the liver express TLRs, including lymphocytes, Kupffer cells, dendritic cells, hepatocytes, hepatic stellate cells, and sinusoidal endothelial cells [10]. TLRs recognize numerous pathogen-associated molecular patterns (PAMPs) *in vitro* and *in vivo* and serve as an important part of the innate immune system. The TLRs recognize bacteria, DNA or RNA, viruses, fungi and protozoa and then activate the immune system to promote the production of inflammatory cytokines and bacitracin that help the body to clear the pathogen and maintain homeostasis [30].

It is well-known that maternal nutrition is critical to the development of human immune system [8]. Animal data suggest that nutritional restriction during gestation shows persistent immunological impairment for several months [9]. He et al. [19] reported that a 40% maternal protein or energy restriction during late gestation decreases the baseline immune status in neonatal goats and alters immune responses to challenges later in life. Equils et al. [13] found that maternal starvation downregulates hepatic *TLR4* expression and function during intrauterine growth in the offspring of prenatal calorie-restricted rats. Therefore, we wondered whether maternal malnutrition affects immune system development especially regarding *TLR* expression in the innate immune system. The liver is an important site of immune precursor cell development and differentiation during early pregnancy [21]. However, the immune status of the fetal liver responding to maternal malnutrition is still unclear. Furthermore, the mother’s system may respond to malnutrition by programming nutrient partitioning to change growth rate and function of the major fetal organs [11]. Hence, how to allocate the limited nutrient partitioning to improve innate immune system function in response to pathogen invasion after birth needs to be investigated.

Therefore, the objective of this study was to assess the immune status of fetal goats in response to maternal feed intake restriction in the middle of the gestation period. Understanding the effects of maternal nutrition on the innate immune system and TLR pathway may potentially help to develop novel strategies to improve the body weight of newborn goats to ensure a higher survival rate.

## Material and methods

The experiments were conducted according to the Animal Care and the Use Guidelines of the Animal Care Committee, Institute of Subtropical Agriculture, Chinese Academy of Sciences, Changsha, China.

### Animal management and dietary treatments

Twelve goats (33.4 ± 7.9 kg at 45 ± 3 d of pregnancy, Xiangdong black goat, local meat breed) were selected and randomly assigned into two groups: the control group [fed 100% of maintenance requirements as reference to Chinese meat goat requirement (2004), *n* = 6] and the restriction group (fed 60% of the control group during d 55 to 100 of gestation, *n* = 6). All animals were penned individually, fed with a 50:50 ratio of concentrate to roughage twice per day (at 08:00 and 16:00), and this protocol lasted from d 45 to 55 of gestation. The feed intake of each experimental goat was recorded during the adaptation period. During the complete restriction period from d 55 to 100 of gestation, feed allowance of the control group was adjusted by 10% upwardly or downwardly per week in order to maintain leftover feed was not higher than 10% and corresponding intake adjustment was performed in the restriction group to maintain 60% intake of the control group. Ingredients and composition of the experimental diet for the pregnant goats are listed in Table 1.

**Table 1:** Ingredients and composition of experimental diets for pregnant Xiangdong black goats, DM basis

Item	Content
Ingredients, %	
*Miscanthus*	54.41
Maize	30.55
Soybean meal	9.41
Fat power	3.65
Calcium carbonate	0.44
Calcium bicarbonate	0.42
Sodium chloride	0.21
Premix^a^	0.91
Composition^b^	
Metabolic energy, MJ/kg	9.11
Crude protein, %	7.19
Ether extract, %	8.97
Neutral detergent fiber, %	64.44
Acid detergent fiber, %	28.32
Ash, %	5.89
Calcium, %	0.97
Phosphorus, %	0.20

^a^Contained per kg: 119 g MgSO_4_•H_2_O, 2.5 g FeSO_4_•7H_2_O, 0.8 g CuSO_4_•5H_2_O, 3 g MnSO_4_•H_2_O, 5 g ZnSO_4_•H_2_O, 10 mg Na_2_SeO_3_ ,40 mg KI ,30 mg CoCl_2_•6H_2_O, 95,000 IU vitamin A, 17,500 IU vitamin D, 18,000 IU vitamin E

^b^Crude protein, ether extract, neutral detergent fiber, acid detergent fiber, ash, calcium and phosphorus were determined values, and metabolic energy was calculated according to the data of Zhang [33]

### Tissue sample collection

All the pregnant goats were anesthetized with an intravenous injection of sodium pentobarbital (50 mg/kg BW) and slaughtered at d 100 of gestation before feeding in the morning. Twelve fetuses (5 males and 7 females) from the control group and twelve fetuses (6 males and 6 females) from the restriction group were collected. At slaughter, the fetal body weight, sex and multiple births were recorded. The heart, liver, spleen, kidney and lung were then weighed immediately. Liver tissues were washed with iced PBS to remove the bloody dirt and then cut into small pieces. Liver samples were immediately frozen in liquid nitrogen and stored at − 80 °C before analysis.

### Blood sampling and analysis

Blood samples were collected by venipuncture from umbilical cord after ligation. A portion of the sample was injected into tubes containing sodium heparin while the remainder of the sample was allowed to coagulate for 40 min before centrifugation (3500×*g*, 10 min). The isolated serum and plasma samples were then stored at − 80 °C until analysis. The examination of plasma albumin (ALB), alkaline phosphatase (ALP) and haptoglobin were performed by automatic biochemistry analyzer (cobas c 311 , Roche). Serum amyloid protein A (SAA), paraoxonase (PON) and IL-6 were assayed using corresponding commercially available ELISA kits (CUSABIO; MEIMIAN). An analysis of amino acid content in blood was performed using an amino acid analyzer (L8800, Hitachi Ltd., Tokyo, Japan). Seventeen individual amino acids were measured. Measured amino acids were divided into essential amino acids and nonessential amino acids.

### Total RNA extraction and qPCR

Total RNA was isolated from liver samples using Trizol (R1100; Solarbio) according to the manufacturer’s protocol. RNA quality was verified by both agarose gel (1%) electrophoresis and spectrometry (A260/A280, NanoDrop 2000; Thermo Scientific.). First-strand cDNA was generated in duplicate using the PrimeScript™ RT reagent Kit with gDNA Eraser (RR047A; Takara). Primers (Table 2) for the target genes and the reference gene were designed and synthesized by Sangon Biotech (Shanghai; China). In the current study, the β-actin gene was chosen as the reference gene. Real-time qPCR was performed in triplicate to amplify the target genes and β-actin using the 2× SYBR Green I PCR mix (Solarbio, SR1110). Reactions were performed in the Roche LightCycler 480II Sequence Detection System. The thermal cycling parameters were as follows: 95 °C for 10 min for activating the hotstart DNA polymerase, and then cycled at 95 °C for 20 s and 60 °C for 1 min for 40 cycles of amplification.

**Table 2:** Primer sequences for the mRNA expression analysis of genes

Gene name	Primers (5′ to 3´)	Length, bp
ActinB (*β-actin*)	F: ATGGCTACTGCTGCGTCGT R: TTGAAGGTGGTCTCGTGGAT	161
Toll-like receptor 2 (*TLR2*)	F: ACGACGCCTTTGTGTCCTAC R: GGGACGAAGTCTCGCTTATG	121
Toll-like receptor 3 (*TLR3*)	F: ATTGGGCAAGAACTCACAGG R: AGGCTTGGAACTGAGGTGAA	119
Toll-like receptor 4 (*TLR4*)	F: ACTCCCTCCCTAGCCTTCAG R: GCCGTGATACGGTTGAAACT	124
Toll-like receptor 7 (*TLR7*)	F: GTTCCATTTCCTTGCACACC R: GGGCACATGCTGAAGAGAGT	123
Myeloid differentiation primary response 88 (*MyD88*)	F: GAGGACGTGCTGATGGAACT R: CGAGGGATGCTGCTGTCTAT	125
Interleukin-1 receptor-associated kinase 1 (*IRAK1*)	F: GACACCGACACCTTCAGCTT R: TGCCTCCTCTTCAACCAAGT	117
TNF receptor associated factor 6 (*TRAF6*)	F: CAGCAGTGCAATGGGATTTA R: CCGGGTTTGCCAGTATAGAA	119
Toll like receptor adaptor molecule 1 (*TRIF*)	F: ACTTCTCACAGGCACCACCT R: GGTCCCTCTCTGATTCCACA	119
TANK-binding kinase 1 (*TBK1*)	F: TGATCACGTTGGATTTCTGC R: GCTTGGTGCGTATGTCTGAA	120
Nuclear factor kappa B subunit 1 (*NFKB1*)	F: GTGCTCGGTGGGAGTAAGAG R: CTCCCGTCACTGCATAGTCA	119
NFKB inhibitor alpha (*NFKB1A*)	F: CTACACCTTGCCTGTGAGCA R: CACGTGTGGCCATTGTAGTT	116
Interferon regulatory factor 3 (*IRF3*)	F: GACCAGCCATGGATCAAGAG R: CAGGTCGACAGTGTTCTCCA	117
Interferon regulatory factor 7 (*IRF7*)	F: TGGCAGCAGATACTGGTGAG R: GAAGATGGTCCTCCAAGCAG	129
Interferon beta (*IFN-β*)	F: CCATCATTGAGCACCTCCTT R: AGGTGAAGATCGGTCGTGTC	118
Interferon gamma (*IFN-γ*)	F: GAACGGCAGCTCTGAGAAAC R: ATTTTGGCGACAGGTCATTC	125
Transforming Growth factor beta 1 (*TGF-β*)	F: GAACTGCTGTGTTCGTCAGC R: TCCAGGCTCCAGATGTAAGG	126
Tumor necrosis factor (*TNF-α*)	F: CCACTGACGGGCTTTACCT R: TGATGGCAGAGAGGATGTTG	141
Interleukin 1 beta (*IL-1β*)	F: AAGCCTCTCCACCTCCTCTC R: TTGTCCCTGATACCCAAGG	114
Interleukin 2 (*IL-2*)	F: GTGAAGTCATTGCTGCTGGA R: GCGTTAACCTTGGGCATGTA	113
Interleukin 4 *(IL-4*)	F: CATCCTCACATCGCAGAAGA R: ACGCCTAAGCTCAATTCCAG	118
Interleukin 6 (*IL-6*)	F: TGACTTCTGCTTTCCCTACCC R: GCCAGTGTCTCCTTGCTGTT	193
Interleukin 10 (*IL-10*)	F: GCTGTTGCCTGGTCTTCCT R: TGTTCAGTTGGTCCTTCATTTG	178

Relative mRNA abundance was determined using the △cycle threshold (△Ct) method [25]. In brief, a △Ct value is the Ct difference between the target gene and the reference gene (△Ct = Ct_target_-Ct_reference_). For each of the target genes, the △△Ct values of all the samples were calculated by subtracting the average △Ct of the corresponding Control/Restriction group. The relative expression ratio of mRNA was calculated by R = 2^–∆∆Ct^.

### Western blot analysis

Immunoblotting for TLR2, TLR4, MyD88, TRIF, IκBα, and phosphorylated IκBα in the liver samples were performed. Briefly, equal amounts of protein (75–130 μg) were subjected to 10% SDS-polyacrylamide gel electrophoresis, and separated proteins were transferred to a polyvinylidene fluoride membrane. The membranes were blocked with 5% nonfat dry milk in tris-buffered saline (TBST) buffer at room temperature for 1 h. Then, the membranes were incubated with Anti-TLR2 antibody (Abcam, ab191458; 1:20,000 dilution), Anti-TLR4 antibody (Abcam, ab13556; 1:1,000 dilution), Anti-MyD88 antibody (SIGMA, SAB2104398-50UG; 1:2,000 dilution), Anti-TRIF antibody (Abcam, ab205363; 1:500 dilution), Anti-IκBα antibody (CST, 9242S; 1:1,000 dilution) and Anti-phosphorylated IκBα antibody (CST, 9246S; 1:1,000 dilution) in TBST including 5% BSA overnight at 4 °C. This was followed by an incubation with horseradish peroxidase-conjugated secondary antibodies (Beyotime, CN) in TBST including 0.1% BSA at 1:1,000 for 1 h at room temperature. The immunoreactive proteins were detected using ECL Prime Western blotting detection reagent by enhanced chemiluminescence. Densitometric analysis was performed with ImageJ 1.42r (National Institutes of Health, USA) software. β-actin was used to normalize the expression of proteins in each sample.

### Statistical analyses

Data were analyzed using the MIXED procedure of IBM SPSS Statistics Version 22. Maternal feed intake restriction and fetal sex were considered fixed effects and the interaction of feed intake restriction × fetal sex was considered in the model. Differences were considered significant at *P* < 0.05, and *P*-values between 0.05 and 0.10 were considered trending towards significance.

## Results

### Dry matter intake and body weight of maternal goats

As shown in Table 3, the actual dry matter intake of pregnant goats in the restriction group was 52.63% of that in the control group (*P* <  0.001). Meanwhile, maternal body weight of the restriction group was lower than that of the control group (*P* <  0.05).

**Table 3:** Dry matter intake and maternal body weight of the Xiangdong black goats during the pregnancy

Item	Control group	Restriction group	SEM	*P*-value
DMI, kg/d	1.14	0.60	0.04	< 0.001
Maternal body weight at d100, kg	39.85	32.14	1.17	0.008

*DMI* dry matter intake

### Fetal body weight, visceral organs development and umbilical cord blood analysis

At d 100 of gestation, fetal body weight showed no difference between the two groups (*P* > 0.05) but the heart weight and index of fetal goats from the restriction group was lower (*P <* 0.05) than that of the control group. However, the weights and organ indices of the liver, spleen, kidney and lung were unaffected (*P >* 0.05) by maternal intake restriction (Table 4). All these parameters were not affected by fetal sex (*P >* 0.05). The interaction between maternal feed intake restriction and fetal sex affected fetal body weight, spleen weight, heart index and spleen index (*P* < 0.05).

**Table 4:** Body weight, organ weights and indices of the fetal Xiangdong black goats during the pregnancy

Item	Control group	Restriction group	SEM	*P*_treatment_	*P*_sex_	*P*_treatment × sex_
Body weight at d 100, g	638.97	564.58	36.04	0.344	0.996	0.041
Heart, g	5.64	4.38	0.24	0.006	0.840	0.246
Liver, g	39.86	34.41	2.74	0.249	0.799	0.188
Spleen, g	0.72	0.66	0.11	0.757	0.950	0.018
Kidney, g	7.25	6.79	0.53	0.978	0.663	0.087
Lung, g	18.37	16.38	1.43	0.501	0.473	0.055
Heart weight: BW, ‰	8.92	7.77	0.23	< 0.001	0.844	0.028
Liver weight: BW, ‰	62.28	61.03	1.67	0.350	0.439	0.296
Spleen weight: BW, ‰	1.09	1.15	0.10	0.217	0.828	0.020
Kidney weight: BW, ‰	11.35	11.95	0.55	0.316	0.454	0.521
Lung weight: BW, ‰	29.08	28.69	1.44	0.906	0.454	0.622

*BW* body weight

As illustrated in Table 5, the concentrations of ALB and PON of umbilical cord blood albumin were not affected (*P >* 0.05) by maternal intake restriction, but the concentrations of ALP and SAA in the restriction group were lower (*P <* 0.05) than those of the control group. In addition, fetal sex affected the ALP concentration (109.43 U/L in females vs 92.50 U/L in males). There was no interaction between maternal feed intake restriction and fetal sex on all these indexes.

**Table 5:** Indexes of the umbilical cord blood in fetal Xiangdong black goats during the pregnancy

Item	Control group	Restriction group	SEM	*P*_treatment_	*P*_sex_	*P*_treatment × sex_
ALB, g/L	20.23	20.80	0.53	0.794	0.142	0.268
ALP, U/L	112.22	84.78	8.75	< 0.001	0.003	0.203
PON, U/L	175.61	178.30	8.83	0.647	0.687	0.532
SAA, g/mL	2189.41	1493.30	144.33	0.037	0.342	0.360

*ALB* albumin, *ALP* alkaline phosphatase, *PON* paraoxonase, *SAA* serum amyloid protein A

When the amino acids of the umbilical cord blood were examined (Table 6), only histidine (*P* = 0.084) had a decreasing tendency, and there were no differences for other individual amino acids, total essential amino acids, total nonessential amino acids, and total amino acids between the control and restriction groups (*P >* 0.05). Additionally, amino acid concentrations were not influenced by fetal sex (*P >* 0.05).

**Table 6:** Amino acids concentration in the umbilical cord blood of fetal Xiangdong black goats

Item	Control group	Restriction group	SEM	*P*_treatment_	*P*_sex_	*P*_treatment × *sex*_
EAA						
Thr	164.27	166.76	23.98	0.884	0.635	0.178
Val	404.41	374.95	49.88	0.524	0.530	0.178
Met	387.77	299.21	60.21	0.233	0.891	0.268
Ile	163.31	175.79	19.01	0.785	0.760	0.177
Leu	302.67	313.81	44.34	0.989	0.765	0.225
Phe	190.08	211.64	31.85	0.776	0.850	0.184
His	118.76	90.70	16.41	0.084	0.229	0.130
Lys	442.71	475.14	69.14	0.874	0.874	0.320
NEAA						
Asp	110.97	84.64	26.94	0.330	0.711	0.325
Ser	1047.16	1151.63	76.93	0.907	0.543	0.140
Glu	634.23	696.91	117.79	0.808	0.869	0.843
Gly	607.13	719.13	68.78	0.324	0.195	0.074
Ala	663.13	491.30	56.65	0.137	0.864	0.346
Cys	77.70	72.85	11.32	0.571	0.668	0.164
Tyr	242.86	265.14	36.27	0.846	0.834	0.149
Arg	188.46	240.39	35.28	0.808	0.911	0.718
Pro	305.33	309.24	37.59	0.434	0.169	0.964
Total EAA	2173.97	2108.00	243.12	0.695	0.858	0.196
Total NEAA	4051.84	3953.70	487.11	0.745	0.572	0.212
Total amino acid	6225.82	6061.71	721.15	0.717	0.655	0.190

*EAA* essential amino acid, *NEAA* Non-essential amino acid

### Expression of TLRs signaling pathway-associated genes in the liver of fetal goats

The mRNA abundance of *TLR2*, *TLR4*, myeloid differentiation primary response 88 (*MyD88*), *TNF* receptor-associated factor 6 (*TRAF6*), nuclear factor kappa B subunit 1 (*NFKB1*), NF-kappa-B inhibitor alpha (*NFKBIA*), *IFN-β, TGF-β*, tumor necrosis factor (*TNF-α*) and *IL-1β* (*P* <  0.05) were increased in the fetal liver of the restriction group (Table 7). The expression of *TLR3* (*P* = 0.099) tended to be higher in the restriction group. In addition, the expression of *TNF-α* (1.91 in females vs 1.10 in males) was affected by fetal sex, and *MyD88* (1.55 in females vs 1.19 in males) and *TRIF* (1.29 in females vs 1.06 in males) transcripts in female fetal goats tended to be greater than that of males. The mRNA expression of *TLR7*, *IRAK1*, *NFKB1*, *TNF-α* and *IL-1β* was also affected by the interaction between maternal feed intake restriction and fetal sex (*P* < 0.05).

**Table 7:** Expression of toll-like receptor signaling pathway genes in fetal Xiangdong black goats liver during the pregnancy

Item	Control group	Restriction group	SEM	*P*_treatment_	*P*_sex_	*P*_treatment × sex_
MyD88-dependent						
TLR2	1.11	1.74	0.161	0.001	0.104	0.160
TLR4	1.06	1.34	0.094	0.003	0.128	0.025
TLR7	1.12	1.06	0.090	0.410	0.510.	0.006
MyD88	1.06	1.61	0.099	< 0.001	0.053	0.642
IRAK1	1.05	1.28	0.096	0.356	0.273	0.029
TRAF6	1.06	1.32	0.093	0.005	0.274	0.282
IRF7	1.14	1.18	0.117	0.697	0.645	0.161
NFKB1	1.08	1.33	0.094	0.008	0.636	0.027
NFKBIA	1.03	1.25	0.045	< 0.001	0.797	0.148
MyD88-independent						
TLR3	1.06	1.15	0.070	0.099	0.671	0.071
TRIF	1.04	1.24	0.079	0.101	0.082	0.451
TBK1	1.05	1.22	0.096	0.106	0.396	0.789
IRF3	1.10	1.36	0.085	0.111	0.402	0.543
Cytokine						
IFN-β	1.08	1.32	0.088	0.034	0.207	0.689
TGF-β	1.04	1.26	0.106	0.019	0.469	0.093
TNF-α	1.00	1.95	0.361	< 0.001	0.004	0.001
IL-1β	1.05	1.36	0.086	< 0.001	0.133	< 0.001

**Fig. 1:**
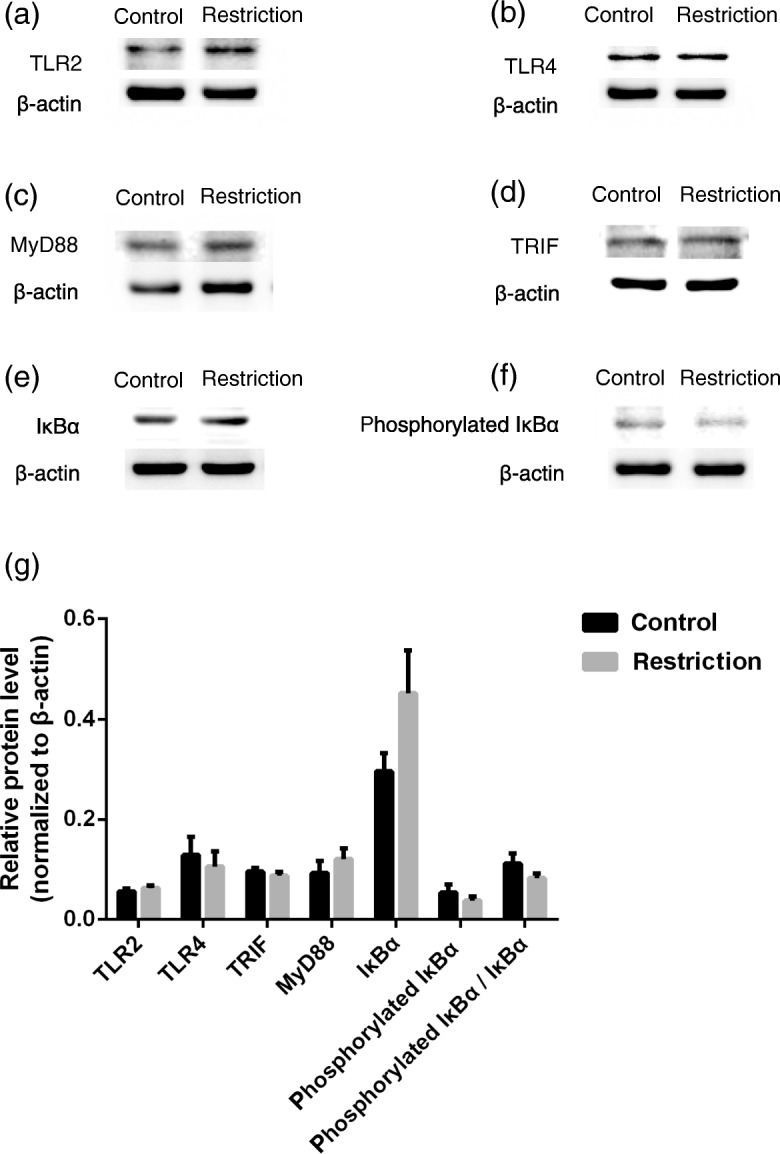
Effects of maternal feed intake restriction on toll-like receptor signaling pathway protein expression of the fetal Xiangdong black goats liver. **a**-**g** TLR2, TLR4, MyD88, TRIF, IκBα and phosphorylated IκBα protein expression in fetal liver analyzed using Western blot. Maternal feed intake restriction and fetal sex did not affect the expression of TLR2, TLR4, MyD88, TRIF, IκBα, phosphorylated IκBα and ratio of phosphorylated IκBα to total IκBα in fetal liver (*P* > 0.05). There was an interaction between maternal feed intake restriction and fetal sex on MyD88 (*P* = 0.025) and phosphorylated IκBα (*P* = 0.018)

To investigate the effects of intake restriction on NF-kappa-B inhibitor alpha (IκBα) expression and activation, the protein expression of IκBα and its phosphorylation at Ser^32/36^ were measured in the liver of fetal goats. As shown in Fig. 1, maternal intake restriction had no effect (*P >* 0.05) on the expression of TLR2, TLR4, TRIF, MyD88, IκBα, and phosphorylated IκBα or on the ratio of phosphorylated IκBα to total IκBα. All of these protein abundances were not affected by fetal sex (*P >* 0.05). There was an interaction between maternal feed intake restriction and fetal sex on MyD88 and phosphorylated IκBα (*P* <  0.05).

## Discussion

The aim of this experiment was to characterize the immune status of fetal livers in response to maternal feed intake restriction during the middle period of gestation. Currently, a 40% intake restriction during the middle period of gestation (d 55–100) in goats was chosen to explore the effects of maternal malnutrition on fetal growth and liver immunity. As a consequence, insufficient nutrient supply inhibited fetal cardiac development during pregnancy and decreased the concentrations of ALP and acute phase protein SAA in the umbilical cord blood. Our results are consistent with previous reports that protein or energy restriction during late gestation altered visceral organ mass [20]. Furthermore, no changes in fetal liver weight between the two groups were observed. It is reported that nutrient restriction during early gestation (from d 30 to d 85) did not influence fetal liver mass in beef cows [11], but maternal undernutrition during late pregnancy (from d 90 to d 140) resulted in inhibited fetal hepatic growth and fibrosis, antioxidant imbalance, and dysfunction in sheep [15]. Generally, ALP plays an integral role in liver metabolism and skeleton development. The ALP activity in female fetuses was greater than that in males in our study. The positive acute phase proteins are regarded as having general functions in opsonization and trapping of microorganisms and their products in modulating the host’s immune response [17]. However, other acute phase proteins (C-reaction protein, haptoglobin and ceruloplasmin) were not detected in the umbilical cord blood of the two groups, we speculate that these acute phase proteins were probably synthesized at a later stage of pregnancy.

Amino acids play critical roles in metabolism, body composition and immunity. Amino acids are required for the synthesis of a variety of specific proteins (cytokines and antibodies) and regulate key metabolic pathways of the immune response to infectious pathogens. In pigs, dietary protein deficiency decreased the umbilical venous plasma concentrations of alanine, arginine, branched-chain amino acids (BCAA; isoleucine, leucine and valine), cystine, glutamine, glycine, lysine, ornithine, proline, taurine and threonine at d 60 of gestation [32]. In ovine, a 50% nutritional restriction between d 28 and d 78 of gestation reduced concentrations of total α-amino acids (particularly serine, arginine-family amino acids, and BCAA) in the fetal plasma [23]. However, in this study, there were no differences in any amino acids between the control and restriction groups. Our results indicate that the mobilization of the protein reserve in pregnant goats fed 60% of maintenance requirements during the middle gestation period appear to sufficiently compensate for the supply of amino acids for the fetuses.

Moderate dietary protein restriction during gestation impairs offspring thymocyte proliferation at birth and thymic and splenic lymphocyte proliferation at weaning in rats [7]. In addition, T lymphocytes from malnourished infants are dysfunctional and have a shorter life [2]. The liver usually contains many immune cells that are responsible for the clearance of foreign antigens, but whether these cells are functionally involved in triggering innate immunity is unclear. Equils et al. [13] have found that a 50% daily food intake restriction from d 11 to 21 of gestation downregulated the hepatic *TLR4* mRNA and protein expression in rats offspring but upregulated hepatic *IL-1β* and *TNF-α* mRNA expression, which have previously been shown to modulate *TLR* gene expression [1]. It was suggested that the effect of intrauterine growth restriction on hepatic *TLR4* expression may be due to the dysregulation of baseline cytokine expression in the liver. In this experiment, we observed the mRNA levels of TLR signaling pathway genes, including *IL-1β* and *TNF-α*, were upregulated in the restriction group. The increase in *TNF-α* mRNA expression in female versus male fetuses needs to be investigated in the future. We further noted the transcripts of *TLR2*, *TLR4*, *MyD88* and *NFKBIA* were upregulated, but protein expression of TLR2, TLR4, MyD88 and total IκBα showed no difference between the two groups. The global reduction in protein translation and subtle changes of transcriptome in the fetal liver of nutrient restricted rats [6] may be ascribed to the differential expression at mRNA and protein levels. Additionally, mature miRNA generally can repress the protein translation process and regulate the abundance of mRNA target genes associated with the innate immune response [22, 31]. Several miRNAs, such as miR-155, miR-146, miR-21, miR-147, miR-9 have been identified to regulate the amplitude of the innate immune response by directly targeting the TLR pathway components or indirectly regulating other networks that cross-talk with TLRs [5, 24, 26, 27, 29]. We surmise that these miRNAs are also probably upregulated by maternal undernutrition and act to repress the TLRs signaling pathway proteins translation process. Furthermore, Ellis et al. [12], using microarray analysis, found that prenatal undernutrition and postnatal leptin treatment led to a limited liver programing, which was associated with increased inflammatory markers and downregulated antigen presentation genes that may lead to immunosuppression. Thus, unchanged hepatic toll-like receptor signaling pathway proteins expression may  also be an appearance of immunosuppression. Certainly, more precise experiments are needed to further verify these processes.

## Conclusion

There were three major findings from this study: 1) maternal feed intake restriction during the middle period of gestation led to an arrested development of the fetuses, especially of the heart; 2) maternal feed intake restriction affected the synthesis of acute phase proteins and alkaline phosphatase; and 3) maternal feed intake restriction mainly regulated the mRNA expression of genes involved in MyD88-dependent signaling pathways and of target cytokines. Further investigations should determine the processes of immune system responses to maternal malnutrition.

## Abbreviations

ALB: Albumin
ALP: Alkaline phosphatase
BCAA: Branched-chain amino acid
IFN-β: Interferon-β
IRAK1: Interleukin-1 receptor-associated kinase 1
IRF3: Interferon regulatory factor 3
IκBα: Inhibitor of nuclear factor kappa-B kinase
MyD88: Myeloid differentiation primary response 88
NFKB1: Nuclear factor kappa B subunit 1
NFKBIA: NFKB inhibitor alpha
PAMPs: Pathogen-associated molecular patterns
PON: Paraoxonase
SAA: Serum amyloid protein A
TGF-β: Transforming growth factor-β
TLRs: Toll-like receptors
TRAF6: TNF receptor associated factor 6
TRIF: Toll like receptor adaptor molecule 1
UCB: Umbilical cord blood
